# Ecological micro-expression recognition characteristics of young adults with subthreshold depression

**DOI:** 10.1371/journal.pone.0216334

**Published:** 2019-05-01

**Authors:** Chuanlin Zhu, Ming Yin, Xinyun Chen, Jianxin Zhang, Dianzhi Liu

**Affiliations:** 1 Department of Psychology, School of Education, Soochow University, Suzhou, Jiangsu, China; 2 Department of Criminal Investigation, Jiangsu Police Institute, Nanjing, Jiangsu, China; 3 School of Humanities, Jiangnan University, Wuxi, Jiangsu, China; National Institutes of Health, UNITED STATES

## Abstract

The micro-expression (ME) processing characteristics of patients with depression has been studied but has not been investigated in people with subthreshold depression. Based on this, by adopting the ecological MEs recognition paradigm, this study aimed to explore ME recognition in people with subthreshold depression. A 4 (background expression: happy, neutral, sad and fearful) × 4 (ME: happy, neutral, sad, and fearful) study was designed; two groups of participants (experimental group with subthreshold depression vs. healthy control group, 32 participants in each group) were asked to complete the ecological ME recognition task, and the corresponding accuracy (ACC) and reaction time (RT) were analyzed. Results: (1) Under different background conditions, recognizing happy MEs had the highest ACC and shortest RT. (2) There was no significant difference in the ACC and RT between experimental and control groups. (3)In different contexts, individuals with subthreshold depression tended to misjudge neutral, sad, and fearful MEs as happy, while neutral MEs were misjudged as sad and fearful. (4) The performance of individuals with subthreshold depression in the ecological ME recognition task were influenced by the type of ME; they showed highest ACC and shortest RT when recognizing happy MEs (vs. the other MEs). Conclusions: (1) The performance of individuals’ ecological ME recognition were influenced by the background expression, and this embodied the need for ecological ME recognition. (2) Individuals with subthreshold depression showed normal ecological ME recognition ability. (3) In terms of misjudgment, individuals with subthreshold depression showed both positive and negative bias, when completing the ecological ME recognition task. (4) Compared with the other MEs, happy MEs showed an advantage recognition effect for individuals with subthreshold depression who completed the ecological ME recognition task.

## 1. Introduction

Micro-expressions (MEs) are often defined as facial movements that are only exhibited for a short time and can reveal emotions that individuals attempt to hide[1, 2]. The duration of expressionsis the main basis for distinguishing between MEs and ordinary facial expressions [3]. Researchers have varying opinions on the ME presentation time. For example, some researchers suggest that the ME presentation time is shorter than 333 ms[4], while others postulate that the total duration of a ME is less than 500 ms or that its initial presentation is less than 260 ms[2]. Since they are automatic real emotional expressions, MEs are more difficult to control than ordinary expressions[5]. Previous studies demonstrated that ME recognition can be used in the clinical field, for example, ME recognition training contributes to improvement in schizophrenics’emotional recognition ability [6, 7]. Additionally, MEs are important cues for detecting deceptive behaviors and revealing true feelings[8].

Ekman and Friesen [9] designed the first standard ME recognition test, i.e., Brief Affect Recognition Test (BART). In this test, participants were asked to view various ME images (happiness, sadness, fear, anger, disgust, and surprise), with presentation times varying between 40 ms and 10 ms, after which they were asked to complete an emotional classification task. The corresponding accuracy (ACC) in detecting the ME is then analyzed. Although this test laid the foundations for follow-up studies, it has some shortcomings. First, the BART lacks ecological validity. Each ME is presented independently, i.e., with no forward and backward expressions, and this does not match the true nature of MEs in real-life [10–12]. Second, it is difficult to measure real ME recognition with this test, since visual aftereffect, which may elongate the processing time of the target stimuli, cannot be avoided. Therefore, Matsumoto et al. [13] developed the Japanese and Caucasian Brief Affect Recognition Test (JACBART). In this test, a neutral expression image (mask stimulus) is presented (2000 ms), followed by a flash of non-neutral facial expression (target stimulus), after which a neutral expression image (mask stimulus) is presented again (2000 ms). The identity of the people in the mask stimuli and target stimulus were controlled. The mask stimulus assists in the elimination of the visual aftereffect of the target stimulus. Participants are asked to judge the emotion conveyed by the target stimuli. Later studies [6, 14] demonstrated that the JACBART has good validity and reliability.

Although the influence of visual aftereffects is controlled in the JACBART, it only tests the influence of neutral background expression (non-emotional expression) to individuals’ ME processing. It does not allow researchers to determine whether there is a difference between the effect of different background expressions on individuals’ performance in completing the ME recognition task. To answer this question, Zhang et al. [15] explored ME recognition (anger, disgust, fear, surprise, and happy), under happy, sad, and neutral background expressions. The results showed that the ACC of recognizing all MEs were lower under sad background expressions, compared to those under happy and neutral background expressions, while the ACC between happy and neutral background expressions showed no significant differences. Zhang et al. [15] upgraded existing research by not only examining the role of neutral background expressions on ME recognition, but also examining the role of positive and negative background expressions on ME recognition. However, they did not examine the influence of different negative background expressions on ME recognition. Based on what’s mentioned above, Zhang et al. [12] compared the participant’s ability to recognize MEs with happy, surprised, angry, disgusting, sad, and fearful background expressions. The results showed that the main effects of the angry, disgusting, sad, and fearful background expressions were significant, while those of happy and surprise were not significant. Based on these findings, Zhang et al. [12] established an ecologically valid ME recognition test. The MEs in the study conducted by Zhang et al.[12] were the so-called ecological MEs, which refer to MEs that occur under different background expressions, rather than those that only occur under neutral ones. ME recognition is a cognitive activity that requires higher sensitivity than ordinary facial expression recognition. Ecological MEs more closely mimics the real-life process.

Previous studies demonstrated that individuals’ performance in recognizing ordinary facial expressions is influenced by age [16], culture [14, 17], and occupation [16]. Additionally, a recent review indicated that one’s facial expression recognition ability is also affected by clinical disease [18]. Several reviews/meta-analyses showed that psychopathological variables may explain the variations in the ACC of recognition of emotional facial expressions. This is especially relevant to depression, since decreased social support, satisfaction, and well-being of interpersonal relationships has been proven to be associated with impaired recognition of facial expressions [19, 20]. Additionally, a recent empirical study [21] showed that a individual’s performance in completing ME recognition task was also influenced by depression. To be more specific, although there was no significant difference in the ACC of recognizing MEs between people with and without depression, the corresponding RTs of those with depression were longer compared to healthy individuals. Individuals with subthreshold depression are those who have clinically relevant depressive symptoms, but do not meet the diagnostic criteria for major depressive disorders [22, 23]. Subthreshold depression is detrimental to individuals’ physical and mental health and is often accompanied by a lower quality of life, higher medical costs [24, 25], high risk of developing depression [26–28], and increased risk of suicide [29–31]. It is important to delineate any existing deficiencies in ecological ME recognition abilities of an individual with subthreshold depression. If there are deficiencies, targeted training could address potentially slower recognition speeds, thereby, improve their interpersonal relationships and well-being. However, few studies have explored the facial expression processing characteristics of individuals with subthreshold depression. The exception being a recent study [32], which mentioned that the role of the depression level should be considered when evaluating an individual’s ME recognition characteristics.

Thus, adopting the ecological ME recognition test established by Zhang et al. [12], the current study aimed to explore the ecological ME recognition characteristics of people with subthreshold depression. Based on previous studies, we hypothesized the following: (1) the ACC and RT of recognizing MEs would be influenced by the background expressions; (2) although there would be no significant difference in the ACC of recognizing ecological MEs between individuals with subthreshold depression and healthy individuals, the RT of the former would be longer than the latter. Additionally, a rapid response to biologically relevant stimuli, for example, snakes, is believed to be evolutionarily significant to us [33, 34]. Numerous studies revealed that both healthy individuals and depressive patients showed negative biases when processing facial expressions[35–38], and a review showed that negative bias is an important risk factor in depression[39]. Thus, we hypothesized that (3) individuals with subthreshold depression would show a negative bias when completing the ecological ME recognition task.

## 2. Materials and methods

### 2.1 Participants

All subjects were recruited from Soochow University, Suzhou, China. The specific steps in selecting theparticipants were proceeded in twostages: (1) 350 undergraduate students were asked to complete the revised Beck Depression Inventory-II (BDI-II)[40], which was proved to be with good reliability and validity[23, 41]. Participants who scored 14 and above or 6 and below were invited toparticipate in the next session, which was conducted approximately 1 week later. (2) in this session, subjects were asked to complete an in-person screening session, whichincluded dministration of the Structured Clinical Interview for DSM-IV-TR Axis I Disorders (SCID)[42] and the BDI-II. The inclusion criteria as following: (1) didn’t fulfil the diagnostic criteria for MDD; (2) had no current schizophrenia, bipolar disorder orpanic disorder; (3) had no concurrent psychotherapy and psychotropic medication.

Finally, 32 subjects (22 female, 18–22 years old) with a BDI score of 14 and above atthe second point in time were assigned to the experimental group, and 32 subjects (22 female, 18–21 years old) with a BDI score of 6 and below were assigned to control group. In addition, we also refer to previous study [21] when determining the sample size. The basic information of both groups is shown in Table 1. All participants were right-handed and normal or corrected to normal eyesight visual acuity. This study was approved by theEthics Committee of Soochow University, which is accordance with the Declaration ofHelsinki (1991). The individual in this manuscript has given written informed consent (as outlined in PLOS consent form) to publish these case details. Participants received 50 RMB for their participation.

**Table 1:** Characteristics of the experimental and control groups.

	experimental group (n = 32) (*M*±*SD*)	control group (n = 32) (*M*±*SD*)	*t*
age	18.72±0.92	18.53±0.92	-0.816
education	12.03±0.18	12.09±0.30	1.025
BDI score	19.63±4.55	6.56±3.92	-12.308***

Note:

"***" stands for "*p*< 0.001". Unit for education: years.

### 2.2 Experimental apparatusand experimental stimuli

A 17-inch Cathode Ray Tube monitor of Tsinghua Tongfang computer was adopted to present the experimental procedure, with a resolution of 1280 × 1024 pixels(refresh rate = 75 Hz).

Forty grayscale images (RGB:127, 127, 127; 338 × 434 pixels) of 10 models (5 females) with facial expressions of sadness, fear, neutral and happiness were selected from Ekman’s Pictures of Facial Affect (POFA) [43]. The reason for choosing those four facial expressions was due to the fact that these expressions have been widely adopted in previous similar studies [11, 15, 21]. The use of these types of expressions can better compare the results of this study with previous studies. The intensity of these images were four, i.e., images with the highest intensity were adopted.

### 2.3 Experimental design and procedure

A 4 (background expression: happy, neutral, sad and fearful) ×4 (ME: happy, neutral, sad and fearful) ×2 (group: individuals with subthreshold depression vs. healthy individuals) study was adopted in this study, with the background expression and ME as within-subject factors, while the group as between-subjects factor. The experimental procedure was written and present by the E-Prime 2.0 software (Psychology Software Tools Inc., Pittsburgh, PA, USA). The experimental procedure was consisted of two stages. In the training phase, 16 practice trials were provided, which was used to ensure subjects fully understood the experimental procedure. Feedback was provided for each trial in the training phase, while no feedback was provided in the formal test. The flowchart of training phase was the same as the form phase. According to previous study [13, 21], the form stage was consisted of 4 blocks, while each block was consisted of 40 trials, a total of 160 trials. Block design has been adopted, only one type of background expression (sadness, fear, neutral and happiness) was adopted in each block. The four blocks were presented in a counterbalanced manner.

**Fig 1:**
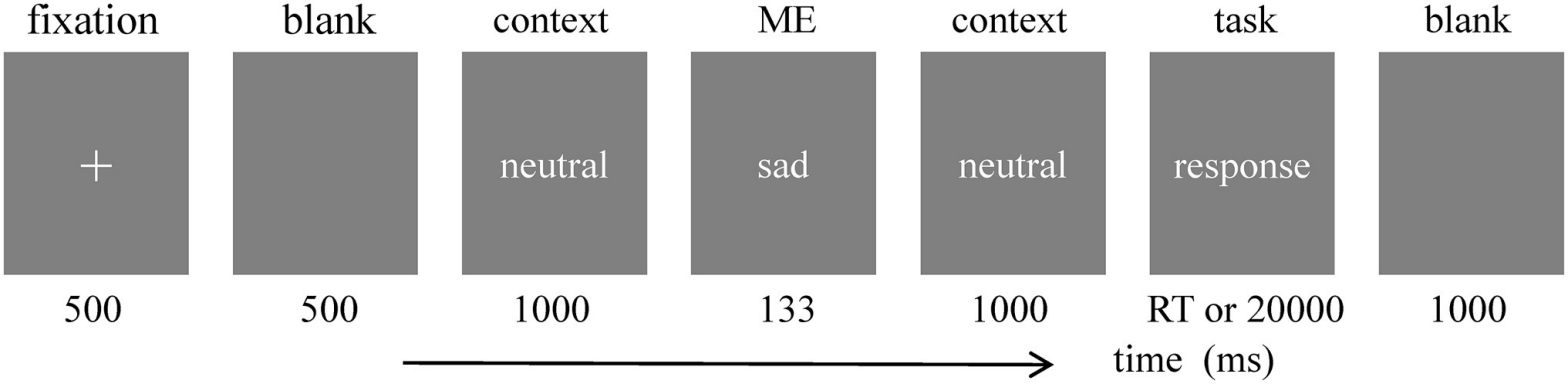
The flow chart of one trial.

As shown in Fig 1, each trial started with white fixation cross (500 ms), followed by a blank (500 ms), the background image (1000 ms), the target expression image (133 ms), next, the same background image (1000 ms). After that, the labels of the four target expressions (sadness, fear, neutral and happiness) were presented, subjects were asked to judge the target expression, then, a blank (1000 ms). Subjects were asked to press the “J” key with their right index finger, if they judge the target expression as sadness, press “K” key with right middle finger when fear, press “D” key with left middle finger when happiness, press “F” key with left index finger when neutral. Key-response were counterbalanced across the trials. Subjects were asked to rest 2 minutes after each block. Subjects were asked to response as accurately as possible, and they have 20000 ms to response at most. The experiment was conducted in a sound-proof room, the participants’ eyes approximately 70 cm from the center of the screen. All stimuli were presented at the centre of the screen. In order to avoid the influence of identify, the images in each trial comes from the same model.

### 2.3 Data recording and analysis

All statistical analysis was performed using SPSS 16.0, post hoc testing was conducted using the Bonferroni correction, while *p* values werecorrected by Greenhouse-Geisser method.

## 3. Results

### 3.1 Indicator 1: The ACC of recognizing MEs

When subjects completed the ecological ME recognition task, four choice (happy, neutral, sad, and fear) were provided, so the chance level was 0.25. The results of one-sample *t*-test showed that the experimental and control group’s ACC of recognizing all MEs were significantly higher (*p*s< 0.001) than 0.25, suggesting that the ACC of recognizing MEs were not the results of random guessing. For the measures of ACC, a 4 (background expression: happy, neutral, sad and fear) × 4 (ME: happy, neutral, sad and fear) × 2 (group: individuals with subthreshold depression vs. healthy individuals) repeated-measures ANOVA was performed, with group as between-subjects factor, and the type of background expression and ME as within-subject factors. The results showed significant main effect of background expression (*F*(3, 186) = 11.64, *p* < 0.001, $\eta_{p}^{2}=0.158$) and ME (*F*(3, 186) = 55.179, *p* < 0.001, $\eta_{p}^{2}=0.471$), while the main effect of group was not significant (*F*(1, 62) = 1.578, *p* = 0.214, $\eta_{p}^{2}=0.025$). Additionally, the interaction effect of background expression with ME was significant (*F*(9, 558) = 27.931, *p* < 0.001, $\eta_{p}^{2}=0.311$), the interaction effect of background expression with group (*F*(3, 186) = 0.253, *p* = 0.859, $\eta_{p}^{2}=0.004$), ME with group (*F*(3, 186) = 0.928, *p* = 0.403, $\eta_{p}^{2}=0.015$), background expression with ME with group (*F*(9, 558) = 0.224, *p* = 0.963, $\eta_{p}^{2}=0.004$) were not significant.

**Fig 2:**
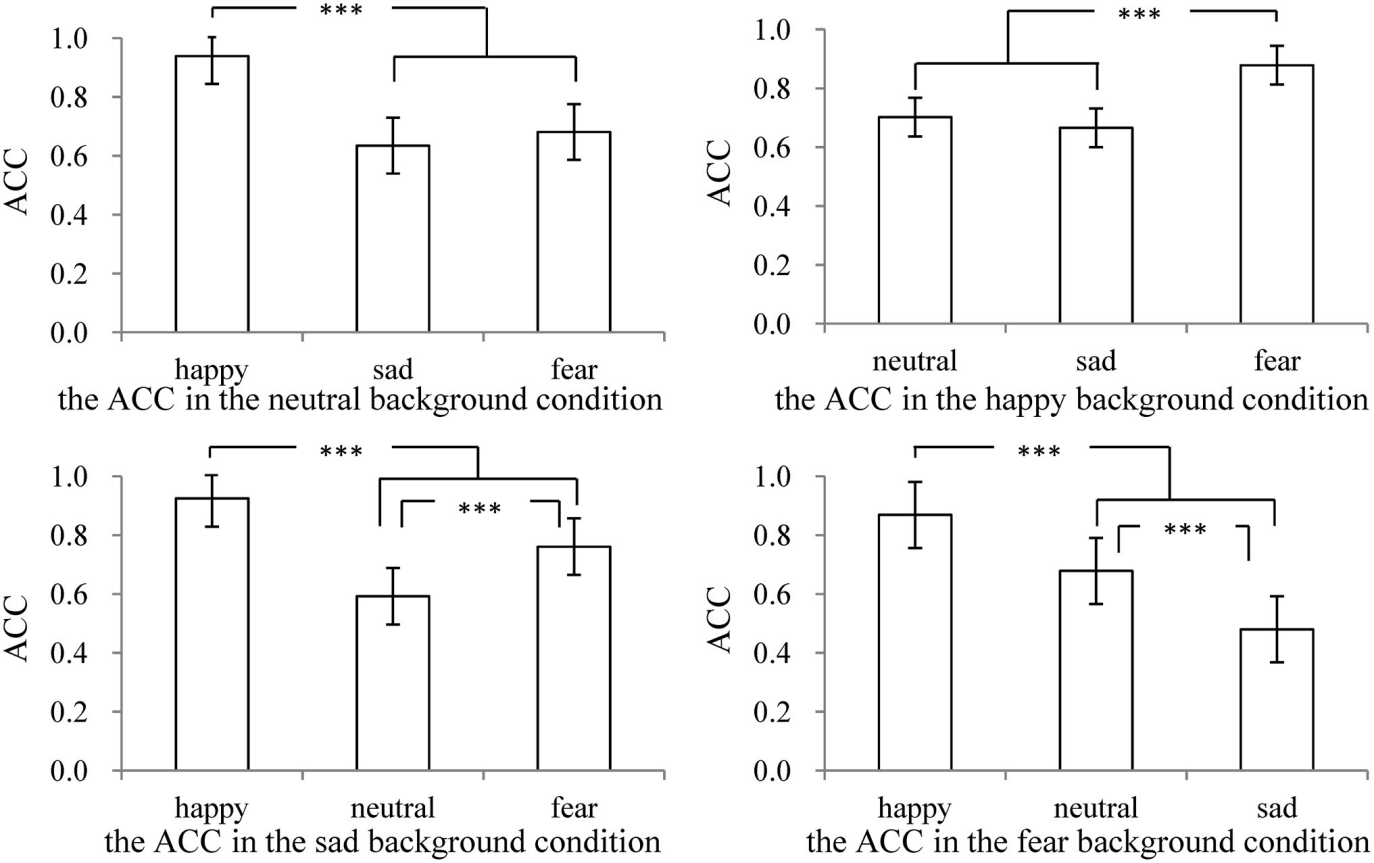
The ACC of recognizing different MEs in different background conditions. “***”stands for “*p*< 0.001”. Error bar represents standard errors.

A simple effect analysis was conducted, since the interaction effect of background expression with ME was significant. The results were as following: (1) In neutral background expression condition, the ACC of recognizing happy and neutral MEs were higher (*p*s< 0.001) than that of recognizing sad and fear MEs. (2) In the happy background expression condition, the ACC of recognizing happy and fear MEs were higher (*p*s< 0.001) than that of recognizing neutral and sad MEs. (3) In sad background expression condition, the ACC of recognizing happy MEs was higher (*p*s< 0.001) than that of recognizing neutral, sad, and fear MEs, and the ACC of recognizing sad and fear MEs were higher (*p*s< 0.001) than recognizing neutral ones. (4) In fear background expression condition, the ACC of recognizing happy MEs was higher (*p*s< 0.001) than that of recognizing neutral and sad MEs, while the ACCs of recognizing neutral and fear MEs were higher (*p*s< 0.001) than that of recognizing sad MEs, and the ACCs of recognizing fear MEs were higher (*p*< 0.001) than that of recognizing neutral MEs. It suggested that individuals’ ACC of recognizing ecological MEs were influenced by the interaction of background expression with ME. The ACC of recognizing happy ME were minimally influenced by the types of background expressions, to be more specific, the ACC of recognizing happy ME was the highest under different background conditions, which also indicated that happy ME was the most easily recognized. The ACC of recognizing other MEs were varied with the change of background expression. See Fig 2 for details.

In conclusion, individual’s ACC of recognizing MEs was significantly influenced by the type of background expression and ME, and the interaction effect of those two factors, while it was not significantly influenced by whether experienced subthreshold depression.

### 3.2 Indicator 2: The RT of recognizing MEs

For the measures of RT of participants correctly identified various MEs, a 4 (background expression: happy, neutral, sad and fear) × 4 (MEs: happy, neutral, sad and fear) × 2 (Group: individuals with subthreshold depression vs. healthy individuals) repeated-measures ANOVA was performed, with group as between-subjects factor, and the type of background expression and ME as within-subject factors. The results showed significant main effect of background expression (*F*(3, 186) = 31.830, *p* < 0.001, $\eta_{p}^{2}=0.339$) and ME (*F*(3, 186) = 24.805, *p* < 0.001, $\eta_{p}^{2}=0.\;286$), while the main effect of group was not significant (*F*(1, 62) = 0.053, *p* = 0.819, $\eta_{p}^{2}=\;0.001$). Additionally, the interaction effect of background expression with ME was significant (*F*(9, 558) = 10.315, *p* < 0.001, $\eta_{p}^{2}=0.143$), the interaction effect of background expression with group (*F*(3, 186) = 1.295, *p* = 0.278, $\eta_{p}^{2}=0.020$), ME with group (*F*(3, 186) = 1.176, *p* = 0.319, $\eta_{p}^{2}=0.019$), background expression with ME with group (*F*(9, 558) = 0.587, *p* = 0.752, $\eta_{p}^{2}=0.009$) were not significant.

**Fig 3:**
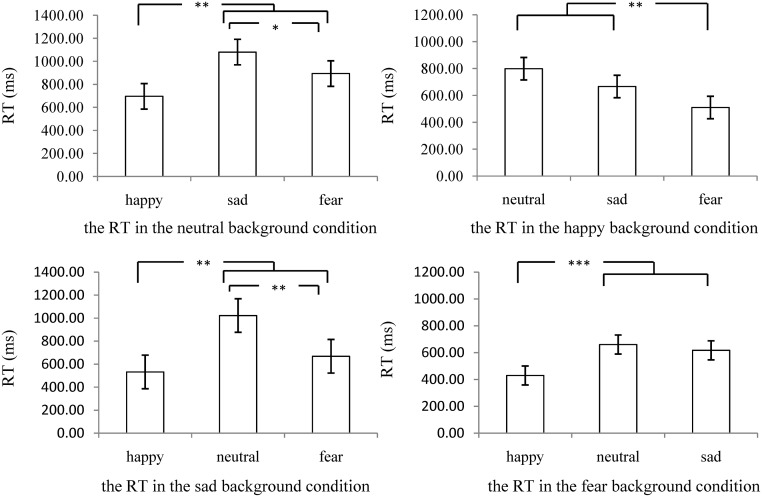
The RT of recognizing different MEs in different background conditions. “*”stands for “*p*< 0.05”, “**”stands for “*p*< 0.01”, “***”stands for “*p*< 0.001”. Error bar represents standard errors.

As the interaction effect between background expression and ME was significant, a simple effect analysis was conducted. The results were as following: (1) In neutral background expression condition, the RT of recognizing neutral (*p*< 0.001), sad (*p*< 0.001), and fear (*p* = 0.004) MEs were longer than that of recognizing happy ones, while the RT of recognizing neutral (*p*< 0.001) and sad (*p* = 0.044) MEs were longer than that of recognizing fear ones. (2) In the happy background expression condition, the RT of recognizing happy (*p*< 0.001), neutral (*p*< 0.001), and sad (*p* = 0.008) MEs were longer than that of recognizing fear MEs. (3) In sad background expression condition, the RT of recognizing neutral (*p*< 0.001), sad (*p*< 0.001), and fear (*p* = 0.002) MEs was longer than that of recognizing happy MEs, and the RT of recognizing neutral and sad MEs were longer (*p*s< 0.001) than recognizing fear ones. (4) In fear background expression condition, the RT of recognizing neutral, sad, and fear MEs was longer (*p*s< 0.001) than that of recognizing happy MEs, the RT of recognizing fear MEs was longer than that of recognizing neutral (*p* = 0.012) and sad (*p* = 0.003) MEs. As shown in Fig 3, in different background expression conditions, individual’s speed of recognizing happy MEs was the fastest, the speed of recognizing fear MEs was medium, while recognizing neutral and sad MEs slowest.

In summary, individual’s speed of recognizing MEs was significantly influenced by the type of background expression and ME, and the interaction effect of those two factors, while it was not significantly influenced by whether experienced subthreshold depression.

### 3.3 Indicator 3: The misjudgment mode of recognizing MEs

Misjudgment refers to judge one ME as the other. Reference to previous research[21], healthy individual’s misjudge mode in neutral background expression condition were chose as baseline, independent sample t-test was conducted, the experimental and control groups’misjudgment mode in happy, sad, and fear background expression conditions was compared with the baseline, respectively. Only when the misjudgment probability in the other conditions, i.e., in happy, sad, and fear background conditions, is significantly higher than the baseline level, can we draw the conclusion that there is a significant difference between baseline and the misjudgment probability of recognizing MEs in the other background expression conditions. The results are shown in Table 2.

**Table 2:** The results of independent sample t-test of experimental and control groups’ misjudgment (*df* = 62).

	group	*M*	*SD*	*t*	*p*
happy—neutral→happy	1	0.034	0.011	3.232	0.003
	2	0.009	0.005	1.791	0.083
happy—neutral→sad	1	0.106	0.041	2.584	0.013
	2	0.134	0.047	2.885	0.006
happy—neutral→fear	1	0.081	0.019	4.314	0.000
	2	0.081	0.021	3.894	0.000
happy—sad→happy	1	0.119	0.018	6.697	0.000
	2	-0.116	0.018	-6.441	0.000
happy—sad→neutral	1	0.166	0.045	3.660	0.001
	2	0.119	0.043	2.785	0.007
happy—fear→happy	1	0.166	0.033	4.949	0.000
	2	-0.181	0.032	-5.620	0.000
sad—happy→fear	1	0.022	0.014	1.542	0.129
	2	0.025	0.012	2.104	0.039
sad—neutral→happy	1	0.125	0.029	4.296	0.000
	2	0.147	0.030	4.824	0.000
sad—neutral→sad	1	0.056	0.028	1.972	0.053
	2	0.081	0.035	2.344	0.023
sad—neutral→fear	1	0.153	0.031	4.952	0.000
	2	0.106	0.025	4.261	0.000
fear—happy→neutral	1	0.081	0.020	3.975	0.000
	2	0.059	0.020	2.972	0.005
fear—neutral→happy	1	1.000	0.021	4.748	0.000
	2	0.100	0.024	4.209	0.000
fear—neutral→sad	1	0.119	0.038	3.101	0.003
	2	0.153	0.044	3.517	0.001
fear—sad→happy	1	0.063	0.025	2.548	0.013
	2	-0.066	0.027	-2.403	0.019
fear—sad→neutral	1	0.353	0.052	6.752	0.000
	2	0.288	0.050	5.765	0.000
fear—sad→fear	1	0.081	0.026	3.129	0.003
	2	-0.088	0.026	-3.405	0.002

Note: “happy—neutral→happy” signifies that participants judged the neutral ME under the happy expression condition as happy. The same applies for the other conditions. M, mean, represents the number of occurences of each error type; SD, standard deviation. Group: "1" stands for experimental group, "2"stands for control group.

As shown in Table 2: (1) the probability of the experimental group misjudge neutral MEs as happy ones, in the happy background condition, were higher than baseline, while there was no significant difference between those of control group and baseline. (2) the probability of the control group misjudge happy MEs as fear ones, in the sad background condition, were higher than baseline, while there was no significant difference between those of experimental group and baseline. Meanwhile, in other conditions, both groups’ misjudgment probability were higher than baseline, but the results of independent sample t-test between the misjudgment probability of experimental and control groups’ showed that, there was no significant difference between those two groups in all conditions (*p*s> 0.05). (3) the experimental group tend to judge happy ME as neutral, during the fear background expression condition, while judge neutral ME as sad/fear, during happy, sad, and fear background expression conditions. In short, both groups’ misjudgment mode were influenced by background expression. Additionally, their misjudgment mode were diverse, i.e., they showed both positive bias and negative bias, positive bias means that both groups tend to judge neutral, sad and fear MEs as happy, while negative means they tend to judge happy as neutral, judge neutral as sad and fear.

## 4. Discussion

Previous studies have focused on comparing characteristics of ordinary facial expression processing in depressive patients and healthy individuals, with few studies comparing the ecological ME recognition characteristics between these two groups. Furthermore, no study has investigated the ecological ME recognition characteristics of individuals with subthreshold depression. Based on the ecological ME recognition paradigm [12], the current study explored the ecological ME recognition characteristics of individuals with subthreshold depression, for the first time.

The current study explored the relationship between background expressions (happy, neutral, sad, and fear) and individuals’ performance in completing ecological ME recognition tasks. The results demonstrated that the participant’s performance was influenced by the type of background expression and MEs. First, the ACC of recognizing MEs differed under different background expressions. For example, there was no significant difference in the ACC of recognizing fearful and sad MEs under a neutral background condition, however, the ACC of recognizing a fearful ME was higher than that of recognizing a sad ME under a happy background condition. This suggested that when studying an individual’s ecological ME recognition characteristics, the influence of different background expressions must also be taken into account. Overlooking the effect of background expressions or only considering the influence of neutral background expression is not appropriate [9, 13]. A large number of studies [44–47] on the processing of ordinary facial expressions have shown that the participant’s performance would be affected by background information. For example, a previous study [48] showed that the N170 amplitudes elicited by the presence of a face in a fearful (vs. neutral) context were larger, which supports the results of the present study to some extent. Second, under different background expressions, individuals were fastest at recognizing happy MEs, intermediate at fearful MEs, and slowest at neutral and sad MEs. This phenomenon is consistent with the happy-face-recognition superiority effect that was revealed by a recent review [49] which suggests that participants were more sensitive to happy MEs than to other MEs. In addition, compared with sad and neutral MEs, fearful MEs can convey potentially threatening information, and these types of expressions are of important evolutionary significance and are more likely to capture attention [50]. This could provide an explanation for the higher effectiveness and speed in processing and recognizing fearful MEs. In conclusion, the current study demonstrated that background expressions affect the ACC and RT of recognizing ecological MEs, and therefore, confirmed our first hypothesis. Hence, the role of different background expressions should be considered in research on ecological MEs.

Moreover, the ACC and RT of recognizing ecological MEs was not only affected by background expressions and MEs separately, but also by the interaction of these two factors. For example, under a happy background expression, the ACC (RT) of recognizing a fearful ME was higher (shorter), compared with recognizing neutral and sad MEs, which suggested that the roles of background expressions and MEs should both be considered synthetically in an ecological ME recognition task. When examining the interaction between background expression and ME, attention should be paid to whether the background expression and ME categories are the same. If the two are inconsistent, the expression recognition task belongs to the ecological ME recognition task (for example, recognizing a happy ME under neutral background expression), otherwise, it is an ordinary expression recognition task (for example, recognizing neutral ME under a neutral background expression). For example, under a happy background expression, the ACC of recognizing a happy ME is higher than that of recognizing neutral and sad MEs. However, the happy expression (target expressions) belongs to ordinary expression, while the neutral and sad are MEs. Thus, the difference mentioned above is not necessarily caused by the interaction between the background expression and ME, but it may be caused by the difference between the ordinary expression and ME.

The ACC and RT of recognizing ecological MEs were not influenced by whether the individual experienced subthreshold depression. There was no significant difference in the ACC of identifying ecological MEs between individuals with subthreshold depression and healthy individuals. This was not only consistent with the features of processing ordinary facial expressions in depressive patients demonstrated by previous researchers [51, 52] but was also corroborated by the characteristics of processing ecological MEs in those patients [21]. There was also no significant difference in the RT between individuals with subthreshold depression and healthy individuals. However, Zhu et al. [21] found that the RT of recognizing ecological MEs in patients with depression was longer than that of healthy individuals. In addition to the participants’ condition (depressive patients vs. individuals with subthreshold depression), the other experimental conditions in the study conducted by Zhu et al. [21] and the current study were the same. Thus, when compared to healthy individuals, individuals with subthreshold depression did not seem to show ecological ME recognition impairment, in other words, their ecological ME recognition ability was relatively normal. Therefore, our second hypothesis was partially confirmed.

Previous studies demonstrated that depressive patients tended to judge happy facial expressions as neutral when recognizing ordinary facial expressions [35, 53, 54]. The third indicator in current study demonstrated that individuals with subthreshold depression tended to judge happy MEs as neutral, under a fearful background expression, which was consistent with previous findings to a certain extent. In addition, compared with the baseline, individuals with subthreshold depression tended to judge neutral MEs as sad/fear, during happy, sad, and fear background expression conditions, indicating a negative bias in the identification of ecological MEs. The present study also demonstrated a positive bias in individuals with subthreshold depression; they tended to judge neutral MEs as happy under a happy background expression. However, in previous studies, depressive patients showed an obvious negative bias when processing facial expressions [35, 54, 55]. This difference could be attributed to three causes. First, the experimental paradigms were different. Although a facial expression processing task was provided in both the previous and present studies, previous studies examined the characteristics of ordinary facial expression processing indepressive patients, the role of background expressions were neglected, and the presentation time of the target stimuli was long. However, the present study examined individuals’ ecological ME recognition characteristics, using the ecological MEs recognition paradigm which considers the influence of different background expressions, and the presentation time of the target stimuli was short. Second, the level of depression in participants were different. In the previous study, the participants were clinically diagnosed as suffering from depression, while those in the present study only showed subthreshold depression and this could have affected the processing of facial expression [56]. Overall, when recognizing ecological MEs, the misjudgment mode of individuals with subthreshold depression showed both negative and positive biases. This result could be further studied in future research and may provide inspiration for clinical treatment. With this, our third hypothesis was confirmed.

Using the ecological ME recognition paradigm, the current study explores the ecological ME recognition characteristics of individuals with subthreshold depression, for the first time. The results demonstrated that background expressions should be fully considered when exploring individuals’ ecological ME recognition characteristics. Additionally, the misjudgment mode of identifying the ecological ME in individuals with subthreshold depression showed both positive and negative biases. This indicated that these groups are sensitive to negative emotions and they may intentionally/unintentionally attempt to inhibit negative emotions. The present study extends the investigation of ME processing from healthy individuals to individuals with subthreshold depression, which contributes to understanding the ecological ME recognition characteristics of different groups. However, this study is just an exploratory behavioral experiment, the corresponding brain mechanism that underlies the ecological ME recognition characteristics of individuals with subthreshold depression is still unclear and future research can adopt the ERP/fMRI technology to explore this issue.

## 5. Conclusion

In the current study, the ecological MEs recognition characteristics of individuals with subthreshold depression was explored for the first time, using an ecological ME recognition paradigm. First, the results demonstrated that the ACC and RT of recognizing ecological MEs were influenced by the types of background expressions, highlighting that it should be fully considered when exploring individuals’ ecological ME recognition characteristics. Second, the ACC and RT of the experimental and control groups showed no significant difference, suggesting that the ecological ME recognition abilities of individuals with subthreshold depression were relatively normal. Additionally, the misjudgment mode of the experimental group showed both negative and positive biases. Finally, the category of ecological ME influenced the ACC and RT, with happy MEs being the easiest to identify (with the highest ACC and lowest RT), revealing that individuals with subthreshold depression were most sensitive to happy MEs.
